# First detection of *Wolbachia* in the New Zealand biota

**DOI:** 10.1371/journal.pone.0195517

**Published:** 2018-04-25

**Authors:** Benjamin Bridgeman, Mary Morgan-Richards, David Wheeler, Steven A. Trewick

**Affiliations:** 1 Ecology Group, College of Sciences, Massey University, Palmerston North, New Zealand; 2 Institute of Fundamental Sciences, Massey University, Palmerston North, New Zealand; Centro de Pesquisas René Rachou, BRAZIL

## Abstract

*Wolbachia* is one of the most widespread intracellular bacteria on earth, estimated to infect between 40 and 66% of arthropod species in most ecosystems that have been surveyed. Their significance rests not only in their vast distribution, but also in their ability to modify the reproductive biology of their hosts, which can ultimately affect genetic diversity and speciation of infected populations. *Wolbachia* has yet to be formally identified in the fauna of New Zealand which has high levels of endemic biodiversity and this represents a gap in our understanding of the global biology of *Wolbachia*. Using High Throughput Sequencing (HTS) of host DNA in conjunction with traditional molecular techniques we identified six endemic Orthoptera species that were positive for *Wolbachia* infection. In addition, short-sequence amplification with *Wolbachia* specific primers applied to New Zealand and introduced invertebrates detected a further 153 individuals positive for *Wolbachia*. From these short-range DNA amplification products sequence data was obtained for the ftsZ gene region from 86 individuals representing 10 host species. Phylogenetic analysis using the sequences obtained in this study reveals that there are two distinct *Wolbachia* bacteria lineages in New Zealand hosts belonging to recognised *Wolbachia* supergroups (A and B). These represent the first described instances of *Wolbachia* in the New Zealand native fauna, including detection in putative parasitoids of infected Orthoptera suggesting a possible transmission path. Our detection of *Wolbachia* infections of New Zealand species provides the opportunity to study local transmission of *Wolbachia* and explore their role in the evolution of New Zealand invertebrates.

## Introduction

The bacterium *Wolbachia* [1,2] is estimated to infect between 40 and 66% of arthropod species worldwide [3–5] making it among the most abundant intracellular bacterial genera. *Wolbachia* is a maternally inherited endosymbiont that can induce a range of host phenotypic responses, including cytoplasmic incompatibility, male death, feminization, and parthenogenesis [6–10]. *Wolbachia* infections can therefore have long-term evolutionary effects on their host lineages, in addition to immediate reproductive modifications, by providing pathways to rapid reproductive isolation and influencing the evolution of sex-determining mechanisms [6,7,9–11]. *Wolbachia* is also being trialled as a biocontrol agent of invasive and disease transmiting insects including medflies [12] and mosquitos as part of the Eliminate Dengue Program [13–15]. As *Wolbachia* can become an obligate parasite of parasitic worms it is also the target of research into antimicrobial drugs by the Anti-Wolbachia Consortium, with the goal of preventing the growth and reproduction of the worms and preventing the diseases they induce [16,17].

*Wolbachia* prevalence differs among species and among populations of the same species, ranging in infection frequency between 30 and 100% of individuals within a population [18,19]. Infection rates are a complex issue not yet well understood, but they are likely to be dynamic, and involve host dispersal and the nature of the host-parasite relationship. For example it has been suggested that the degree of infection may be a result of *Wolbachia* acting as a mutualistic secondary symbiont rather than an exclusive reproduction parasite [20–22].

The mechanism(s) by which *Wolbachia* moves between host populations has yet to be confirmed, but is unlikely to be solely via vertical transmission. Genetic similarity of *Wolbachia* found in parasitoids and parasitoid hosts suggest horizontal transmission [23–25]. It has been shown that microinjection of *Wolbachia* infected cells can facilitate transfer [26], and this indicates how *Wolbachia* might be transferred by ovipositing parasitoids. Should the parasitoid egg fail to develop, *Wolbachia* may move in to the host and persist into further generations. Alternatively, the bacterium may be transferred through the digestive system of invertebrates feeding on *Wolbachia* infected hosts as in some other endoparasites (e.g. Gordian worms). Horizontal transmission via the digestive track has been shown to be effective in whiteflies. *Wolbachia* was observed to persist in leaves for up to 50 days, which if fed upon by un-infected whiteflies, resulted in *Wolbachia* infections in the majority of whiteflies [27].

Phylogenetic studies have identified 16 globally distributed supergroups of *Wolbachia* [10,28–32]. Incongruence between *Wolbachia* and host phylogenies suggests many episodes of horizontal transfer resulting in unrelated hosts in the same region sharing similar strains of *Wolbachia* [25,33]. However, inference of phylogenetic relationships is also complicated by recombination among *Wolbachia* strains [28,34,35], and host–parasite coevolution [36]. For this reason, a Multilocus Sequence Typing (MLST) system is now widely used as it allows differentiation between even closely related strains of *Wolbachia* [37].

The New Zealand invertebrate fauna has many distinctive features including high levels of species endemicity [38]. As a large continental island, physically isolated from neighbouring terrestrial ecosystems for many millions of years, the biota has had opportunities to evolve in novel ways and it is frequently posited that the biota has been strongly influenced by their ancient isolation [39,40]. If so, this predicts that distinctive species interactions could have evolved including unique strains of endosymbionts such as *Wolbachia*. However, to date no *Wolbachia* infections have been reported from any New Zealand native invertebrate species, reflecting few targeted attempts at their discovery. We tested whether *Wolbachia* could be detected and if so whether there was evidence of distinctive evolutionary lineages in endemic New Zealand insects.

## Methods

Two different approaches were employed to survey potential hosts for *Wolbachia* infection; bioinformatics and molecular ecology. The first approach made use of bioinformatic tools to search for evidence of *Wolbachia* ‘contamination’ in High Throughput Sequencing (HTS) data (reads and assembled contigs) from various insects. These low coverage DNA sequence datasets were produced to infer molecular phylogenies of the invertebrate species using multicopy markers (e.g. [41,42]. The second approach used the MLST primer sets [37] to search for evidence of *Wolbachia* in a wide range of target templates representing multiple host species and populations.

### Mining next generation DNA sequences

As part of a phylogenomic study of endemic New Zealand Orthoptera that have distinctive regional diversity, we carried out HTS of genomic DNA isolated from members of three families; Acrididae, Anostostomatidae, and Rhaphidophoridae (Table 1). The DNA libraries were sequenced on one lane of an Illumina HiSeq2000 by BGI [43]. Approximately 1–4 Gigabytes of 100bp paired end sequencing data was generated for each of the 21-sequenced species.

**Table 1:** Abundance of *Wolbachia*-like sequence reads in HTS from endemic New Zealand Orthoptera.

Order	Family	*HTS Specimen*	Location	Reads
**Orthoptera**	Rhaphidophoridae	*Macropathus* sp.	Waitomo	30817
**Orthoptera**	Anostostomatidae	*Hemiandrus brucei*	South Island	17220
**Orthoptera**	Rhaphidophoridae	*Talitropsis sedilloti*	Mohi Bush, Hawkes Bay	2486
**Orthoptera**	Rhaphidophoridae	*Miotopus diversus*	Waioeka Gorge, Gisborne	2384
**Orthoptera**	Rhaphidophoridae	*Neonetus* sp.1	Mohi Bush, Hawkes Bay	1363
**Orthoptera**	Rhaphidophoridae	*Neonetus* sp.1	Hongi’s Track, Rotorua	1346
**Orthoptera**	Rhaphidophoridae	*Isoplectron* sp.	Canterbury	154
**Orthoptera**	Rhaphidophoridae	Cave weta	Denniston	126
**Orthoptera**	Rhaphidophoridae	Cave weta	Kapiti Island	47
**Orthoptera**	Rhaphidophoridae	*Macropathus* sp.	Westport	25
**Orthoptera**	Anostostomatidae	*Hemiandrus focalis*	Lake Taupo	21
**Orthoptera**	Anostostomatidae	*Hemideina crassidens*	South Island	20
**Orthoptera**	Rhaphidophoridae	*Pharmacus chapmani*	Old Man Range, Otago	17
**Orthoptera**	Acrididae	*Sigaus australis*	Lindis Pass, South Island	0
**Orthoptera**	Rhaphidophoridae	*Novoplectron serratum*	Chatham Island	0
**Orthoptera**	Rhaphidophoridae	*Pachyrhamma sp*.	Balls Clearing, Hawkes Bay	0
**Orthoptera**	Anostostomatidae	*Hemideina thoracica*	Manawatu	0
**Orthoptera**	Anostostomatidae	*Hemiandrus* sp.	New Zealand	0
**Orthoptera**	Anostostomatidae	*Hemideina crassidens*	North Island	0
**Orthoptera**	Anostostomatidae	*Motuweta riparia*	North Island	0
**Orthoptera**	Anostostomatidae	*Hemiandrus pallitarsis*	Manawatu	0

High Throughput Sequence samples used with location, number of sequences matching *Wolbachia*, and the relative abundance of *Wolbachia*-like sequences detected (Rank).

HTS data was analysed with PAUDA [44], and MEGAN5 [45] in order to identify *Wolbachia* sequences found within each HTS dataset. MEGAN5 [45] visually displays what organisms are detected in the HTS datasets and indicates the number of sequences associated with each species. If *Wolbachia* matches were among the 16 most frequently recorded organisms detected at the level genus, the respective invertebrate host sample was treated as positive for *Wolbachia* infection.

*Wolbachia* sequences from the positive samples were extracted and mapped against the genome of the *Wolbachia* endosymbiont of *Drosophila melanogaster* (accession number NC002978) using Geneious. v. 6 (http://www.geneious.com) [46]. Mapping was performed using the medium sensitivity setting, which equates to a minimum overlap of 25bp, at least 80% overlap identity, with a maximum of 30% mismatches allowed per read. Mapping was iterated five times using the consensus sequence of the reads and repeating the mapping process. This allows more reads to be mapped to variable regions or regions that differ from the reference sequence and reduces the likelihood of reads mapping incorrectly.

Once sequences were aligned to the reference genome, coverage at the five core gene loci of the MLST system (ftsZ, coxA, fpbA, hpcA, gatB [37]) was determined, by identifying the conserved PCR primer binding sites on the reference gene. Targeting primer binding sites allowed direct comparison between the HTS sequences and those obtained via PCR. Where there was sufficient HTS coverage the consensus sequence of mapped reads at MLST loci was included in subsequent phylogenetic analysis.

### Extraction and amplification of *Wolbachia* from invertebrate DNA

We focused our host sampling on multiple individuals of the Orthopteran genus *Hemiandrus* (Family Anostostomatidae) that had yielded positive results in HTS analysis and for which we had suitable material[47]. DNA was extracted from leg or abdomen tissue of 204 individual *Hemiandrus* ground weta. We used a modified salting-out method incorporating an ice-cold ethanol washing step before addition of room temperature ethanol and allowing the ethanol to evaporate, leaving the DNA to be eluted in 50μl water[48]. Extracted DNA was tested for the presence of *Wolbachia* DNA using the MLST primer combinations (Table 2). The 204 individuals represented 16 taxa (*H*. *nox*, *H*. *bilobatus*, *H*. ‘disparalis’, *H*. *electra*, *H*. ‘elegans’, *H*. *focalis*, *H*. ‘furoviarius’, *H*. ‘horomaka’, *H*. *maculifrons*, *H*. *brucei*, *H*. *luna*, *H*. *nitaweta*, *H*. ‘onokis’, *H*. ‘promontorius’, *H*. *subantarcticus*, and *H*. ‘vicinus’), with *H*. *nox* represented by individuals from North Island and South Island [49].

**Table 2:** Summary of New Zealand invertebrate samples tested for *Wolbachia* infection through PCR.

Order	Identification	Total n	Positive n	Infected %	Sequenced n
**Orthoptera**	*Hemiandrus brucei*	89	65	73	53
**Orthoptera**	*Hemiandrus luna*	29	27	93	18
**Orthoptera**	*Hemiandrus maculifrons*	63	36	57	6
**Orthoptera**	*Hemiandrus nox*	10	3	30	2
Orthoptera	*Isoplectron ssp*.	6	3	50	2
**Orthoptera**	*Pachyrhamma ssp*.	39	13	33	1
**Orthoptera**	*Hemiandrus* ‘paturau’	1	0	0	0
**Orthoptera**	*Hemiandrus bilobatus*	1	0	0	0
**Orthoptera**	*Hemiandrus* ‘disparalis’	1	0	0	0
**Orthoptera**	*Hemiandrus electra*	1	0	0	0
**Orthoptera**	*Hemiandrus* ‘elegans’	1	0	0	0
**Orthoptera**	*Hemiandrus focalis*	1	0	0	0
**Orthoptera**	*Hemiandrus* ‘furoviarius’	1	0	0	0
**Orthoptera**	*Hemiandrus* ‘horomaka’	1	0	0	0
**Orthoptera**	*Hemiandrus nitaweta*	1	1	100	0
**Orthoptera**	*Hemiandrus* ‘onokis’	1	0	0	0
**Orthoptera**	*Hemiandrus ‘promontorius’*	1	0	0	0
**Orthoptera**	*Hemiandrus subantarcticus*	1	0	0	0
**Orthoptera**	*Hemiandrus* ‘vicinus’	1	0	0	0
**Hymenoptera**	*Archaeoteleia gilbertae*	3	2	66	2
**Hymenoptera**	*Archaeoteleia onamata*	3	0	0	0
**Hymenoptera**	*Archaeoteleia karere*	3	2	66	1
**Psocoptera**	*Ectopsocus* sp.	2	2	100	1
**Diptera**	*Chlorops* sp.	1	1	100	0
**Lepidoptera**	*Aenetus virescens*	1	0	0	0
**Hymenoptera**	*Vespula vulgaris*	2	0	0	0
**Hymenoptera**	*Vespula germanica*	2	0	0	0
**Hemiptera**	*Scolypopa australis*	2	0	0	0
**Plecoptera**	*Stenoperla* sp.	1	0	0	0
**Coleoptera**	*Halmus chalybus*	2	0	0	0
**Hymenoptera**	*Proctotrupoidea* sp.	1	0	0	0
**Diptera**	*Musca domestica*	1	0	0	0
**Lepidoptera**	*Danaus plexippus*	1	0	0	0
**Diptera**	*Tipulidae*	1	0	0	0
**Diptera**	*Chrysomya rufifacies*	1	0	0	0
***Diptera***	*Fannia canicularis*	1	0	0	0
***Diptera***	*Drosophila* sp.	1	0	0	0
**Diptera**	*Leptotarsus* sp.	4	0	0	0
**Hymenoptera**	*Apsis mellifera*	2	0	0	0
**Diptera**	*Trigonospila brevifacies*	1	0	0	0
**Ephemeroptera**	*Coloburiscus humeralis*	2	0	0	0
**Trichoptera**	*Aoteapsyche* sp.	2	0	0	0
**Hemiptera**	*Siphanta acuta*	2	0	0	0
**Megaloptera**	*Archichauliodes* sp.	2	0	0	0
**Isopoda**	*Ligia novaezealandiae*	1	0	0	0
**Euonychophora**	*Peripatoides morgani*	3	0	0	0

Number of individuals of each species tested, number positive, and number successfully sequenced

*Wolbachia* infection rates in species and populations was tested using PCR targeting 5 MLST loci [37], and the variable *WSP* locus that has potential for distinguishing *Wolbachia* lineages [11,50]. In addition to the absence of *Wolbachia* in a sample, several technical issues could explain false negatives where amplification failed. Therefore, we used positive PCR controls with universal insect mitochondria primers LCO1490 and HCO2198 [51] to target host DNA. DNA from *Nasonia vitripennis* wasps known to be infected with *Wolbachia* was used to verify the specificity of the MLST primers used in this study.

We also searched for *Wolbachia* infection in DNA from 45 Rhaphidophoridae (cave weta) from the genera *Pachyrhamma* (n = 39) and *Isoplectron* (n = 6). To increase taxonomic range, we screened 40 individuals of 24 other invertebrate species (Table 2). Sixteen of these were exotic species and eight were New Zealand native or endemic panarthropoda species. We included nine samples of the parasitoid wasp *Archaeoteleia* because Rhaphidophoridae are their hosts [52], and this parasitic interaction is a potential means of horizontal transmission of *Wolbachia*. A subset of individuals that produced a DNA fragment for the *Wolbachia* ftsZ region were sequenced using the forward ftsZ [37] primer (Macrogen Inc., Korea). DNA sequences were checked for quality and aligned to published *Wolbachia* sequences (Table 3) and our sequences extracted from the HTS samples (Table 1) using Geneious v. 6 [46].

**Table 3:** Published representative *Wolbachia* genome diversity by host taxon.

GenBank *Wolbachia* host	GenBank ID
*Diabrotica barberi* clone	KC578107
*Altica lythri* isolate	KF163343.1
*Pheidole vallicola*	EU127749
*Altica helianthemi*	KF163366.1
*Altica palustris*	KF163363.1
*Altica impressicollis*	KF163368.1
*Altica impressicollis*	KF163367.1
*Drosophila innubila*	EU126333
*Polistes dominulus*	EU126353
*Precis iphita*	FJ392398.1
*Jalmenus evagoras*	FJ392417.1
*Lissorhoptrus oryzophilus*	DQ256473.1
*Wolbachia* sp.	AJ130717.1
*Bombyx mandarina*	KJ659910.1
*Cydia fagiglandana*	KJ140034
*Bryobia kissophila*	JN572863.1
*Bryobia praetiosa*	EU499322.1
*Wolbachia pipientis*	JN316217.1
*Mesaphorura italica*	AJ575103.1
*Altica oleracea*	KF163332.1
*Melittobia digitata*	EU170117.1
*Altica oleracea*	KF163325.1
*Altica oleracea*	KF163324.1
*Serritermes serrifer*	DQ837193.1
*Cubitermes* sp.	DQ127295.1

**Fig 1:**
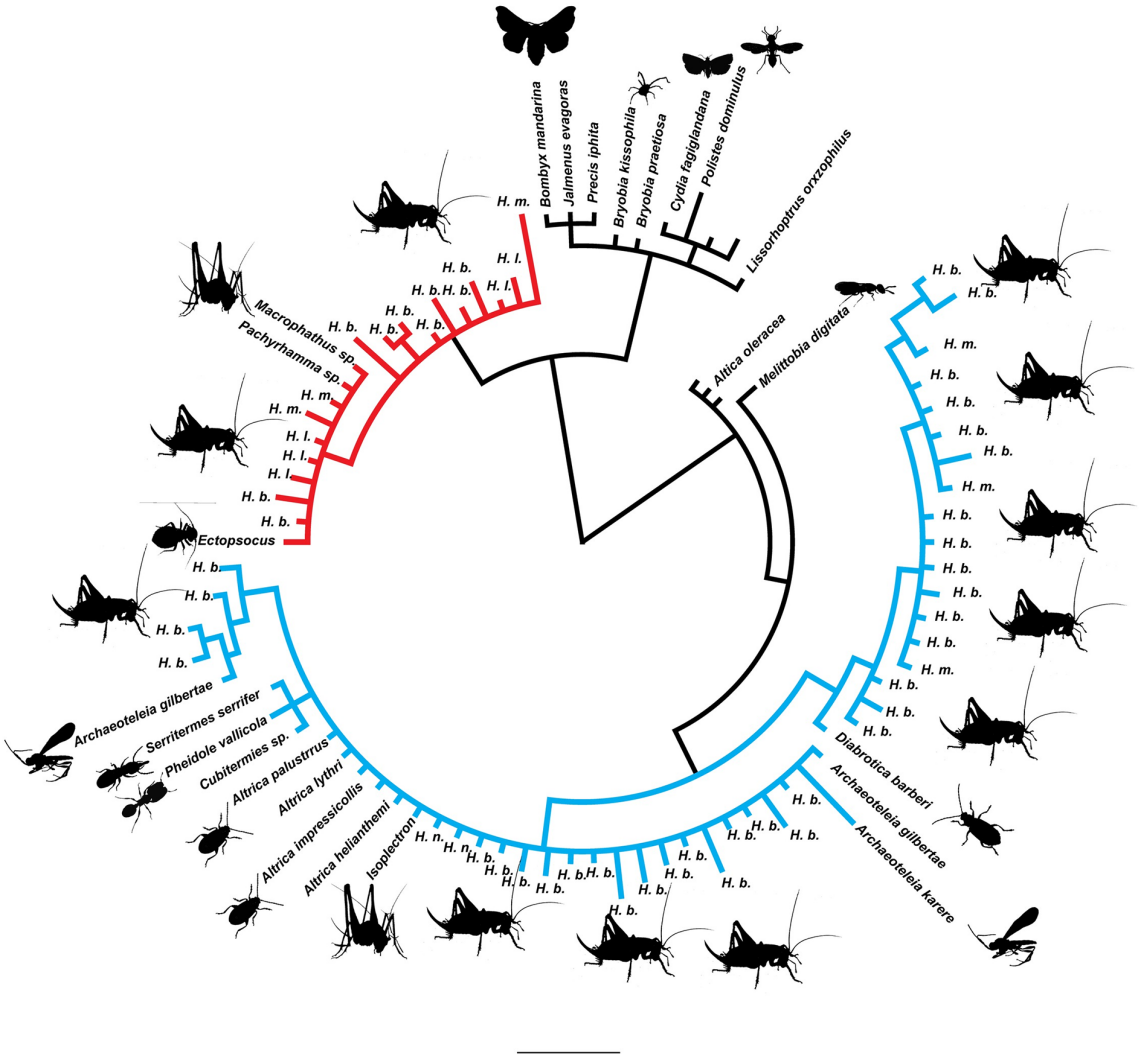
The diversity of *Wolbachia* infections detected in New Zealand illustrated in a phylogeny of novel and published *Wolbachia* DNA sequences at the ftsZ locus (211–438 bp). Species names are those of the hosts. *Wolbachia* supergroup A is in blue, and supergroup B in red. H.b *Hemiandrus brucei*, H.l *Hemiandrus luna*, H.m *Hemiandrus maculifrons*, H.n *Hemiandrus nox*.

**Fig 2:**
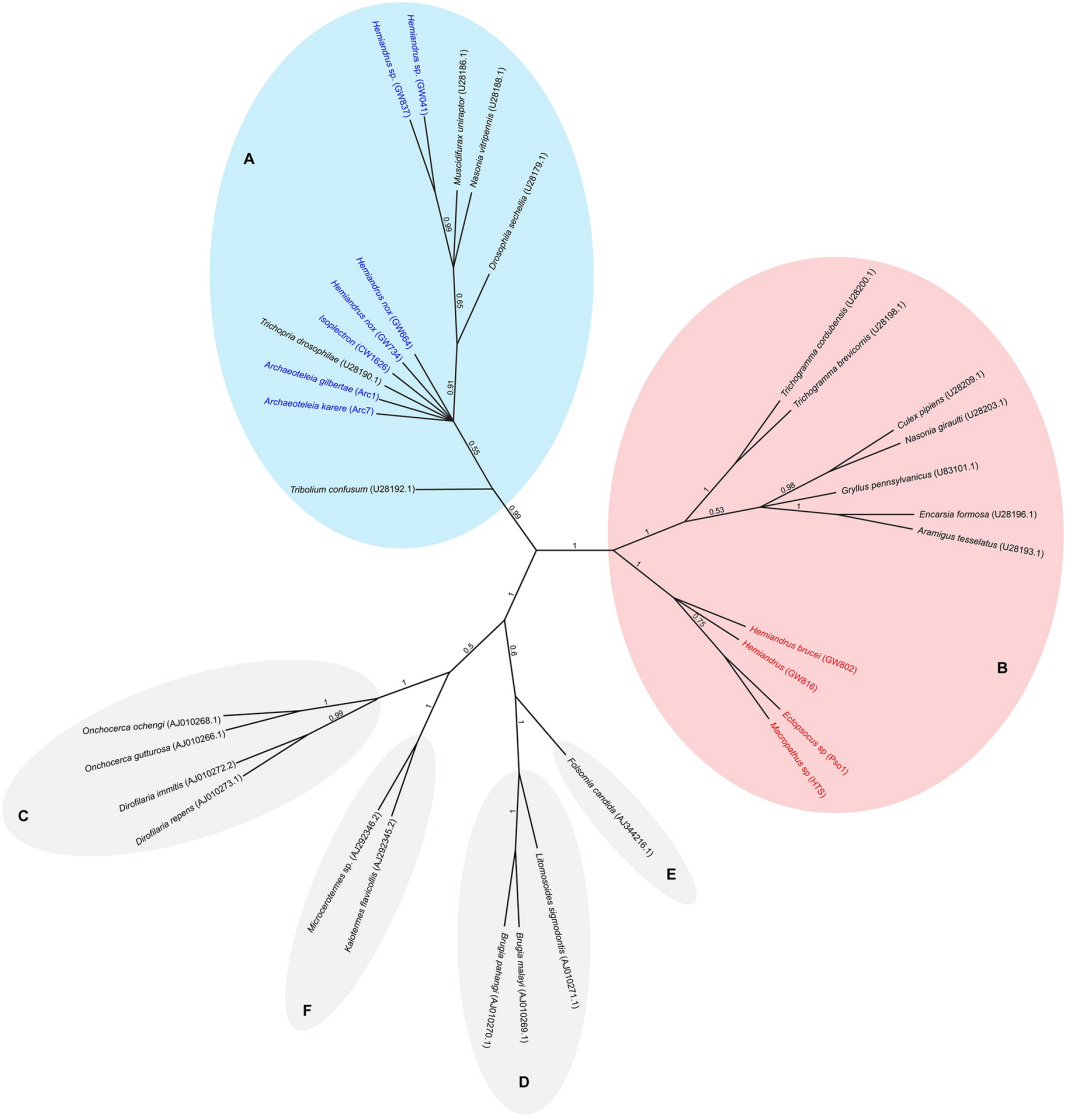
Bayesian phylogenetic analysis of New Zealand and representatives of published *Wolbachia* endosymbionts based on ftsZ sequences. Species names are those of the host of *Wolbachia*. *Wolbachia* supergroups are indicated by the corresponding letter (A–F). Host names in color (blue/red) are insect species endemic to New Zealand.

**Fig 3:**
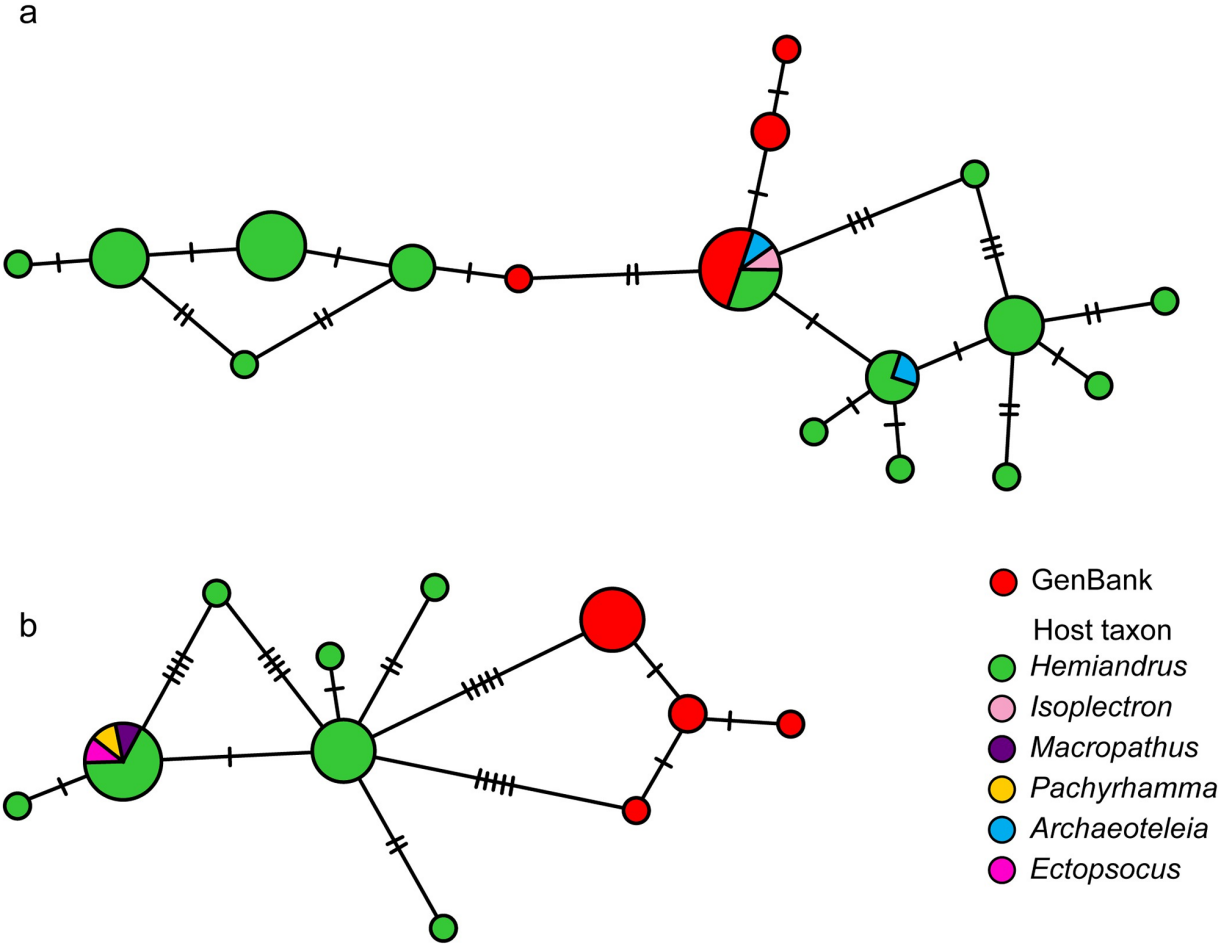
Evolutionary relationships among ftsZ DNA sequences from *Wolbachia* infections of New Zealand insects inferred using minimum spanning networks. Published sequences from similar *Wolbachia* collected outside of New Zealand are coded in red. A. Supergroup (isolate) A *Wolbachia* (360 bp) infecting New Zealand orthoptera and parasitoid wasps. B. Supergroup (isolate) B *Wolbachia* (228 bp) infecting orthoptera and book lice. Numbers of nucleotide differences among FtsZ sequences are indicated.

Eighty-six *Wolbachia* DNA sequences were aligned and trimmed to produce an alignment of 211–438bp of the ftsZ locus. Phylogenetic relationships were inferred using Bayesian phylogenetic analysis (MrBayes; HKY, chain length 1100000, subsampling frequency 200, burn-in length 100000, random 22500) [53,54](Fig 1). Incorporating a published dataset [28] with a subset of data from the present survey (n = 11) allowed us to determine which supergroup the New Zealand *Wolbachia* sequences were most similar to (Fig 2). Minimum spanning network [55] (epsilon 0) analysis was performed using PopART [56] (Fig 3).

**Fig 4:**
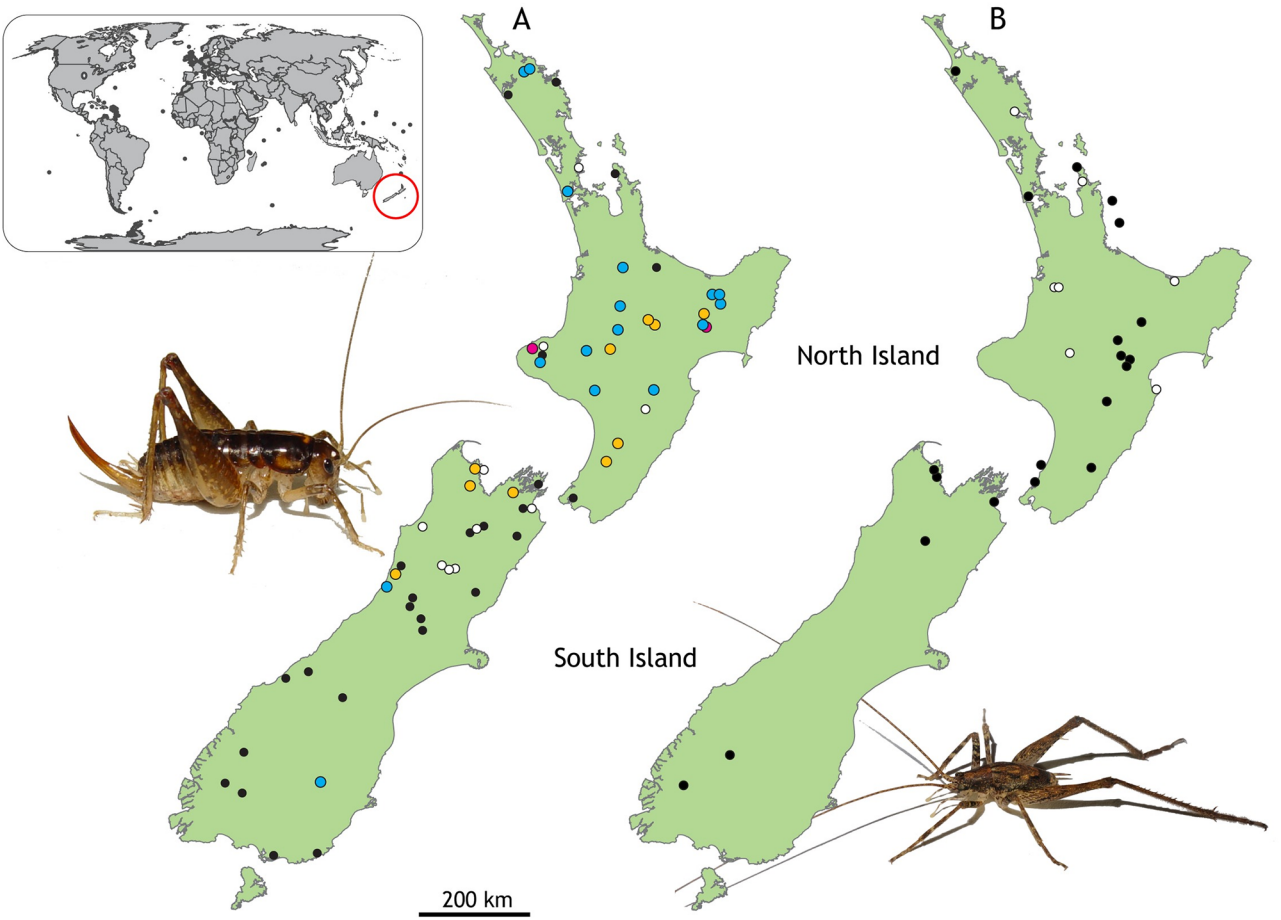
Location and *Wolbachia* infection status of two native orthopteran lineages in New Zealand. A. Collection locations of *Hemiandrus* individuals indicating *Wolbachia* isolate found; Isolate A blue, Isolate B orange, infected but not sequenced white, not infected black, populations containing both Isolate A and Isolate B red. B. Collection locations of Rhaphidophoridae (cave weta) indicating *Wolbachia* infection status; infected white, not infected black. Inset images, female *Hemiandrus brucei* (left) and *Isoplectron armatum* (right).

To determine the spatial distribution of the newly discovered *Wolbachia* infections in New Zealand, ground weta and cave weta collection locations were mapped using QGIS (QGIS Development Team, 2015). Individual locations were coloured according to whether the insects collected there were infected with *Wolbachia* or not (Fig 4). Isolate information was processed through the QGIS dataset to display the distribution of the hosts found carrying each isolate and determine if hosts of differing isolates were likely to be found in sympatry or allopatry.

## Results

### High throughput DNA sequencing of New Zealand Orthoptera

High throughput DNA sequence data was generated for 21 species of New Zealand Orthoptera. Using the metagenomics tools PAUDA and MEGAN to search for evidence of *Wolbachia* sequences within the unassembled insect shotgun sequencing data, six species were found to have *Wolbachia* infections (Table 1). We found similar high levels of *Wolbachia* DNA sequence in a cave weta (*Macropathus* sp.) and a ground weta (*Hemiandrus brucei*). To ascertain the level of genome coverage represented by *Wolbachia* reads in these two species, the short reads were mapped to the complete genome of the *Wolbachia* endosymbiont of *Drosophila melanogaster*. This revealed that the *Macropathus* sp. reads covered 30% of this reference genome, whilst the reads from *Hemiandrus brucei* mapped over 33.6% of the *Wolbachia* reference. Average pairwise nucleotide similarities between the reference *Wolbachia* genome and reads from *Macropathus* sp. and *Hemiandrus brucei* were 90.2% and 92%, respectively. However, due to uneven coverage across the genome only one of the MLST genes (ftsZ) had sufficient sequence information to recover its complete consensus sequence from the HTS data. Four other host species samples that contained significant levels of *Wolbachia* DNA were all endemic Rhaphidophoridae (cave weta), but read numbers were lower compared to those isolated from the *Macropathus* sp. and *Hemiandrus brucei* specimens (<2500 DNA sequences) (Table 1). Low read number corresponded with lower coverage of the *Wolbachia* genome (<5%) when mapped against the reference.

### *Wolbachia* infections using specific primers for DNA amplification

To further explore the degree of *Wolbachia* infection in New Zealand insects we carried out a PCR screen using primers that target *Wolbachia* MLST or *WSP* loci [37]. Positive results for *Wolbachia* infections from six independent Orthoptera lineages were detected through PCR. *Wolbachia* was detected in a clade of Anostostomatidae ground weta (*Hemiandrus brucei*, *Hemiandrus luna*, *Hemiandrus maculifrons* and *Hemiandrus nox)* and in the rhaphidophorid genera *Pachyrhamma* and *Isoplectron* (Table 2).

### Infection rates

Infections rates varied from 30%– 93% of individuals per species where > individuals were tested (Table 2). For Orthoptera species with the largest sample size infection rates were 73% (*H*. *brucei*, n = 89), 93% (*H*. *luna*, n = 29), and 57% (*H*. *maculifrons*, n = 63) positive for amplification using at least one of the MLST primer pairs. *Wolbachia* was detected in three of four individuals of North Island *Hemiandrus nox* but absent from the six individuals from the South Island *Hemiandrus nox* (n = 10) (Table 2). In addition, of a further 12 *Hemiandrus* species tested only a single *Hemiandrus nitaweta* individual (Table 2) gave a positive result.

A total of 45 individual cave weta were tested for the presence of *Wolbachia* from the genera *Pachyrhamma* and *Isoplectron*. Three of the six (50%) *Isoplectron*, and 13 of the 39 *Pachyrhamma* (33%) samples tested positive for *Wolbachia* at one or more of the *Wolbachia* specific primers (Table 2).

As a potential vector for *Wolbachia* horizontal transmission in Rhaphidophoridae and possibly other insects, the parasitoid wasp *Archaeoteleia* was tested for infection. Of nine specimens available for testing, four individuals were positive for an infection; two *A*. *gilbertae* and an individual each of *A*. *onamata* and *A*. *kawere* (Table 2). Of the further 40 invertebrate individuals representing twenty-four species collected from New Zealand and tested for *Wolbachia* using MLST primers few were positive. *Wolbachia* infection was identified in two species; a native tree-living booklouse *Ectopsocus* sp. and an exotic grass fly *Chlorops* sp.

*Wolbachia* infections in New Zealand Orthoptera are geographically widespread (Fig 4). At many locations individuals with *Wolbachia* infections were collected alongside individuals that were not infected with *Wolbachia*, suggesting that where *Wolbachia* is present it is not at saturation. *Wolbachia* was detected throughout the North Island and northern South Island (Fig 4), however, *Wolbachia* was detected at a lower frequency in *Hemiandrus* ground weta from the southern half of South Island, where only two individuals were positive for a *Wolbachia* infection (Fig 4). The *Wolbachia* infection rate in Rhaphidophoridae was highest in samples from central and northern North Island although fewer southern samples were examined (Fig 4).

We sequenced the ftsZ region of *Wolbachia* infections from 82 Orthoptera hosts, three parasitoid wasps (*Archaeoteleia* sp.) and one booklouse (*Ectopsocus sp*.; Table 2). Incorporating representatives of all major global *Wolbachia* supergroups into a phylogenetic analysis with the New Zealand *Wolbachia*, DNA sequences revealed that the New Zealand diversity nested within supergroups A and B, based on the ftsZ gene (Fig 2). The New Zealand *Wolbachia* sequences from *Macropathus* and *Pachyrhamma* cave weta, *Ectopsocus* booklouse and some *Hemiandrus* ground weta fell within supergroup (clade) B, while the *Wolbachia* sequences from *Isoplectron* cave weta and other *Hemiandrus* ground weta fell within supergroup (clade) A. We refer tentatively to New Zealand *Wolbachia* samples that are part of supergroup A [28] as isolate A as we currently have DNA sequence from one locus. Isolate A samples included 43 sequences from New Zealand *Wolbachia*, but the New Zealand representatives did not form a monophyletic group within clade A. However, New Zealand clade B sequences differed from all available published (GenBank) *Wolbachia* (Table 3)(Fig 1) by a minimum of five substitutions (Fig 3) and formed a monophyletic cluster. The closest match was the sister clade consisting of infections from China, India, and Europe [28] and we tentatively referred to as isolate B. Six host individuals had DNA sequences from both isolates, suggesting that they were infected with two different *Wolbachia* lineages.

Minimum spanning networks of *ftsZ* for isolate A (360 bp) and isolate B (228 bp) reveal the diversity within New Zealand *Wolbachia* (Fig 3). Fourteen distinct sequences were identified within isolate A, differing by 1–3 nucleotides. The parasitoid wasp *Archaeoteleia* was infected with *Wolbachia* having the same sequence as that obtained from three different New Zealand orthopteran host species. Seven distinct sequences were identified within isolate B *Wolbachia*, and these differed by a minimum of five mutations from published *Wolbachia* sequences (2.2%; Fig 3).

## Discussion

*Wolbachia* was detected in HTS DNA sequence datasets from six orthopteran individuals that are endemic to New Zealand, representing two families and five genera (*Macropathus* sp., *Hemiandrus brucei*, *Talitropsis sedilloti*, *Miotopus diversus*, and two *Neonetus* specimens). Orthoptera elsewhere in the world are known to be hosts of *Wolbachia* [57,58], but we present the first documented cases of *Wolbachia* infection of any endemic New Zealand invertebrate. The samples from cave weta *Macropathus sp*. (Rhaphidophoridae) and ground weta *Hemiandrus brucei* (Anostostomatidae) provided ~30% coverage of the *Wolbachia* genome which was the largest in our sample. These sequences were unambiguously identified as part of the *Wolbachia* global diversity.

The *Macropathus* sp, and *Hemiandrus brucei* samples yielded approximately twice the number of total DNA reads compared to the other HTS datasets analysed, however, *Wolbachia* was also detected in a number of other Rhaphidophoridae (cave weta) samples. The level of detection in four samples was lower (<4%), but *Wolbachia* represented the majority of prokaryote reads detected in the analysis and DNA sequences were close matches to published *Wolbachia* (Pairwise % Identity and identical sites of the samples of ≥85% in *M*. *diverus* and both *Neonetus* specimens). In contrast, the sample from *Talitropsis sedilloti* had bacterial sequences with less similarity to *Wolbachia* (pairwise 54% and identical 27.1%) which might represent different bacteria.

### *Wolbachia* and the *Hemiandrus maculifrons*-complex

The genomic DNA sequence datasets provided evidence of infection from single representatives of five different species (Fig 4). To investigate infection rates, we amplified DNA from numerous individuals of the same species using specific primers, targeting host species within the same genus. *Wolbachia* was detected in all three species of the ground weta species *H*. *brucei*, *H*. *luna*, and *H*. *maculifrons* through the MLST protocol. Infections were detected in the majority of individuals tested, with 73% of *H*. *brucei*, 93% of *H*. *luna*, and 57% of *H*. *maculifrons*. *Hemiandrus brucei* and *H*. *luna* showed the high-level pattern of infection as suggested by Hilgenboecker, et al. [3]. Their metaanalysis indicates that intraspecific *Wolbachia* infections rates tend to show a ‘most or few’ infection pattern, as very high or very low infection frequencies were more likely to occur than intermediate rates. *Hemiandrus maculifrons* had a lower infection rate, well below the high (>90%) infection level but much higher than in low level (<10%) infections. Within the same host, *Wolbachia* infections can vary among tissue types, tending to be at higher density in female reproductive tissue. Our weta DNA extractions were mostly from femur muscle and as numbers of intracellular bacteria tend to be limited in somatic tissue this may have reduced detectability in our sample [10,34]. Small host sample sizes also make estimates of infection rates less precise, and this can be rectified by expanded sampling now that the Wolbachia target has been recognised. The same *Wolbachia* strain can produce various reproductive modifications (pathenogenisis, male killing, cytoplasmic incombatibility) in different host lineages [10], that result in dissimilar infection frequency [7]. In the morphologicaly cryptic *Hemiandrus* species we studied their genetic similarity suggests it unlikely that *Wolbachia* has caused different reproductive modifications in each species, but further research will reveal if *Wolbachia* was a contributing factor in their speciation.

### *Wolbachia* and Rhaphidophoridae

New Zealand has a high diversity of Rhaphidophoridae (cave crickets or cave weta) with at least 19 endemic genera. The orthopteran family is found worldwide and typically cave-dwelling, but several New Zealand species are unusual in that they inhabit forests and the sub-alpine zones. *Wolbachia* was detected infecting six of these genera; *Pachyrhamma*, *Isoplectron*, *Neonetus*, *Talitropsis*, *Miotopus* and *Macropathus*, with the highest infection rate 33% in *Pachyrhamma*. As we did not target specific tissue known to have high *Wolbachia* densities (ovarian follicles) this might underestimate the true infection rate. At least one species of *Isoplectron* was host to *Wolbachia* with an infection rate detected of 50%. Further samples will need to be tested to determine the level of infection at both the population and species level. *Wolbachia* was detected in *Macropathus* through HTS. Inclusion of this genus in further surveys would be informative.

### Transmission

Intracellular bacteria such as *Wolbachia* are regularly transmitted in egg cells from mother to offspring (vertical transmission [34]). However, *Wolbachia* is also suspected to be transmitted between species horizontally [25,28,34,59], potentially by an uninfected insect eating an infected one or by multiple species being host to the same parasitoid wasps [60,61]. *Archaeoteleia* is a genus of parasitoid wasp known for its parasitism of eggs of New Zealand *Pachyrhamma* cave weta species. However, the typical hosts of two species (*A*. *gilbertae*, *A*. *karere*) that were positive for *Wolbachia* is not known. *Wolbachia* was found in four individuals representing two parasitoid wasp species. The congruence between the *Wolbachia* infecting weta and the *Wolbachia* infecting *Archaeoteleia* may indicate an avenue for further research into a potential interspecies transmission route of *Wolbachia* in weta. Notably, the *Wolbachia* DNA sequences from both *Archaeoteleia gilbertae* and *A*. *karere* were identical to *Wolbachia* sequences from the cave weta *Isoplectron* (not the *Pachyrhamma* examined) and two ground weta species (Anostostomatidae: *Hemiandrus*). The presence of matching *Wolbachia* in *Isoplectron* and ground weta rather than *Pachyrhamma* is interesting because if it is determined that *Wolbachia* can be transmitted via *Archaeoteleia* this may be the first indication of new hosts for these *parasitoids*.

Within the New Zealand insect hosts examined, two distinct clades of *Wolbachia* were detected. Both isolates of *Wolbachia* have managed to infect the New Zealand Rhaphidophoridae. The New Zealand *Wolbachia* lineage that is part of A supergroup clustered with identical *Wolbachia* DNA sequences from hosts sampled outside of New Zealand (Fig 1). In contrast, other New Zealand *Wolbachia* ftsZ gene sequences formed a monophyletic group within supergroup B (Fig 1). This might represent a distinct New Zealand lineage of *Wolbachia*. Further testing of the MLST regions is required because recombination of MLST fragments between strains of *Wolbachia* occurs. The distribution of ‘isolate B’ through *Hemiandrus* sister species was extensive with at least 11 confirmed *H*. *luna* hosts and three confirmed *H*. *maculifrons* hosts in addition to the 14 confirmed *H*. *brucei* hosts. We also detected that some insect hosts were infected with both A and B isolates of *Wolbachia*.

To our knowledge, this work documents the first cases of *Wolbachia* infection in endemic New Zealand insects. We detected infection by *Wolbachia* in endemic species of two families of Orthoptera and in endemic parasitic wasps that attack these Orthoptera. Relatively high observed infections rates, considering our sampling of somatic tissue, in more than one *Hemiandrus* lineage suggest that *Wolbachia* is not involved in formation of reproductive barriers between ground weta species, and no definitive pattern of *Wolbachia* distribution has yet been determined in New Zealand. It was present in all the *Hemiandrus* species tested spanning both main islands. Further study including analysis of female reproductive tissue will inform on the prevalence of infections across the country and among related species, and reveal what, if any, effect, infections have on reproductive capabilities of the endemic New Zealand insect fauna.
