# Correction: First detection of *Wolbachia* in the New Zealand biota

**DOI:** 10.1371/journal.pone.0201151

**Published:** 2018-07-18

**Authors:** The PLOS ONE Staff

There are errors in the Funding section. The correct funding information is as follows: No specific funding was received for this work.

The publisher apologizes for this error.

## References

[1] Bridgeman B, Morgan-Richards M, Wheeler D, Trewick SA (2018) First detection of *Wolbachia* in the New Zealand biota. PLoS ONE 13(4): e0195517 10.1371/journal.pone.0195517 29694414 PMC5918756

